# A Novel Inflammatory-Related Gene Signature Based Model for Risk Stratification and Prognosis Prediction in Lung Adenocarcinoma

**DOI:** 10.3389/fgene.2021.798131

**Published:** 2022-01-05

**Authors:** Wen-Yu Zhai, Fang-Fang Duan, Si Chen, Jun-Ye Wang, Yao-Bin Lin, Yi-Zhi Wang, Bing-Yu Rao, Ze-Rui Zhao, Hao Long

**Affiliations:** ^1^ State Key Laboratory of Oncology in Southern China, Department of Thoracic Surgery, Collaborative Innovation Center for Cancer Medicine, Sun Yat-Sen University Cancer Center, Guangzhou, China; ^2^ Lung Cancer Research Center, Sun Yat-Sen University, Guangzhou, China; ^3^ State Key Laboratory of Oncology in Southern China, Department of Medical Oncology, Collaborative Innovation Center for Cancer Medicine, Sun Yat-Sen University Cancer Center, Guangzhou, China

**Keywords:** lung adenocarcinoma, inflammatory, gene, prognostic signature, risk stratification

## Abstract

Inflammation is an important hallmark of cancer and plays a role in both neogenesis and tumor development. Despite this, inflammatory-related genes (IRGs) remain to be poorly studied in lung adenocarcinoma (LUAD). We aim to explore the prognostic value of IRGs for LUAD and construct an IRG-based prognosis signature. The transcriptomic profiles and clinicopathological information of patients with LUAD were obtained from The Cancer Genome Atlas (TCGA) and the Gene Expression Omnibus (GEO). Least absolute shrinkage and selection operator (LASSO) analysis and multivariate Cox regression were applied in the TCGA set to generate an IRG risk signature. LUAD cases with from the GSE31210 and GSE30219 datasets were used to validate the predictive ability of the signature. Analysis of the TCGA cohort revealed a five-IRG risk signature consisting of EREG, GPC3, IL7R, LAMP3, and NMUR1. This signature was used to divide patients into two risk groups with different survival rates. Multivariate Cox regression analysis verified that the risk score from the five-IRG signature negatively correlated with patient outcome. A nomogram was developed using the IRG risk signature and stage, with C-index values of 0.687 (95% CI: 0.644–0.730) in the TCGA training cohort, 0.678 (95% CI: 0.586–0.771) in GSE30219 cohort, and 0.656 (95% CI: 0.571–0.740) in GSE30219 cohort. Calibration curves were consistent between the actual and the predicted overall survival. The immune infiltration analysis in the TCGA training cohort and two GEO validation cohorts showed a distinctly differentiated immune cell infiltration landscape between the two risk groups. The IRG risk signature for LUAD can be used to predict patient prognosis and guide individual treatment. This risk signature is also a potential biomarker of immunotherapy.

## Introduction

Lung cancer is the second most common malignancy and has the highest mortality rate (Sung et al., 2021). Lung adenocarcinoma (LUAD) is its major histological type, accounting for more than 40% of all lung cancer cases (Lortet-Tieulent et al., 2014; Abe and Tanaka, 2016). Advancements in diagnostic and therapeutic strategies, especially those in molecular targeted therapy and immunotherapy, have done much to improve the outcomes of LUAD (Steven et al., 2016; Vargas and Harris, 2016); however, patient survival rates remain low (Zhao et al., 2018). The existing tumor-node-metastasis (TNM) system accounts for only a few factors and neglects heterogeneity in molecular characteristics (Balachandran et al., 2015). Thus, it is necessary to develop new prognostic biomarkers for LUAD to identify heterogeneous patients who are candidates for individual antineoplastic therapy.

Inflammation is the immune system’s response to infection, trauma, and other stresses (Gomes et al., 2014). It is also important in cancer, where it plays multiple roles (Hanahan and Weinberg, 2011). On the one hand, the local inflammatory response promotes the occurrence and development of tumors by releasing important molecules and carcinogens to the tumor microenvironment. These can include survival factors that limit apoptosis, growth factors that sustain the proliferative signaling, and pro-angiogenic factors (Ben-Baruch, 2006; Hanahan and Weinberg, 2011). On the other hand, the lymphocyte-to-monocyte ratio and the neutrophil-to-lymphocyte ratio, which are indicators of the systemic inflammatory response, and have been found to be prognostic factors in patients with LUAD (Takahashi et al., 2016; Minami et al., 2018).

Several studies have reported the prognostic value of different inflammatory-related genes (IRGs). For example, He Z et al. found that BTG Anti-Proliferation Factor 2 (BTG2) expression can suppress the proliferation and metastasis of NSCLC cells (He et al., 2015). Recently, the potential prognostic value of IRGs has also been explored in colorectal cancer, low-grade glioma, and oral cavity squamous cell carcinoma (Bai et al., 2019; Liang et al., 2021; Xiang et al., 2021).

The predictive ability of a multi-gene model is superior to that of a single-gene model (Srivastava and Gopal-Srivastava, 2002). In this study, we used data from The Cancer Genome Atlas (TCGA) to isolate an IRG-based signature associated with the survival rates of LUAD patients. Data from the Gene Expression Omnibus (GEO) was then used to validate the predictive ability of this signature. Finally, a nomogram was developed using the aforementioned IRG-based signature to more precisely predict the outcome of LUAD patients.

## Materials and Methods

### Data Collection and Preparation

RNA-seq data and clinical information of patients with LUAD were downloaded from the TCGA (https://tcga-data.nci.nih.gov/tcga/) and GEO databases (https://www.ncbi.nlm.nih.gov/geo/). Cases with incomplete clinical information and follow-ups of less than 5 days were excluded. Finally, 488 cases from the TCGA database were used as the training cohort while 226 cases from the GSE31210, and 85 cases in GSE30219 datasets were used as the validation cohort. The different gene expression datasets were normalized using the “limma” and “SVA” R packages. The Masked Somatic Mutation data (varscan. Somatic. Maf) was analyzed using the “maftools” R package (Mayakonda and Koeffler, 2016). Finally, IRGs were selected and downloaded from hallmark gene sets in the Molecular Signatures Database (http://www.gseamsigdb.org/gsea/msigdb/cards/HALLMARK_INFLAMMATORY_RESPONSE.html). These are shown in Supplementary Table S1.

### Construction and Validation of the Prognostic IRG Signature

Differentially expressed genes (DEGs) were defined as having false discovery rates (FDR) < 0.05 and log2 |fold change| > 2. Differential expression analysis between LUAD tumor tissues and para-carcinoma tissues was performed using the “limma” R package. In the training cohort, the differentially expressed IRGs were first subjected to univariate Cox regression analysis (*p* < 0.05). Following this, the least absolute shrinkage and selection operator (LASSO) regression analysis was performed to narrow down the prognostically significant candidate IRGs. Then, multivariate Cox regression analysis was used to determine the best weighting coefficient of each prognostically significant candidate IRG. This IRG signature included all the differentially expressed and prognostically significant IRGs. The risk score for each case was calculated according to normalized expression levels of IRGs and their corresponding regression coefficients following the same kind literature (Huang, et al., 2021). The specific formula for this is as follows: Risk score = sum (expression level of each IRG × corresponding coefficients).

In the TCGA training cohort, all patients were divided into a high-risk and low-risk group according to the cut-off value of risk scores derived from maximally selected log-rank statistics, which was used the R package “Maxstat” with the Horton and Lausen (HL) *p* value approximation method (Hothorn and Zeileis 2008)The Kaplan-Meier method was used to compute the overall survival (OS), and the log-rank test was used to compare OS between the two groups.

To validate the IRG signature, the risk score of LUAD cases in the GSE31210, and GSE30219 datasets were calculated using the same formula as the TCGA cohort. Cases in the validation set were also divided into two groups according to the cut-off point of risk score obtained from the maximally selected log-rank statistics. Survival curves of the low- and the high-risk groups in the validation cohort were also estimated using the Kaplan-Meier method and were compared via the log-rank test.

### Gene Set Enrichment Analyses

To investigate the potential molecular mechanisms of the signature IRGs that were identified, Gene Set Enrichment Analysis (GSEA) was used to find enriched terms between the high-risk and low-risk patients in the TCGA and GEO cohort. After excluded gene with no expression value, all mRNA was entered in GSEA which performed in Java GSEA v. 4.0.1 with the Kyoto Encyclopedia of Genes and Genomes (KEGG) pathway in C2, and Gene Ontology (GO) terms in C5. After performing 1,000 permutations, genes with a false discovery rate q < 0.05 were deemed significantly enriched.

### Immune Cell Infiltration and Tumor Mutation Burden Analyses

After normalizing the expression data in the TCGA and GEO dataset, we used a single sample GSEA (ssGSEA) to evaluate 28 immune cells using the R package “GSVA” (Tamborero et al., 2018). The results were visualized in a heatmap. With the perm set to 1,000, the CIBERSORT software package was used to perform the CIBERSORT algorithm and evaluate the proportion of 22 types of infiltrating immune cells based on LM22 (Newman et al., 2015). In TCGA cohort, according to the length of the human exon, the TMB calculated for each patient was calculated as the total mutation frequency/35 MB. Dividing the total number of mutations by the size of the coding region of the target is the resulting TMB per megabase. The Mann-Whitney U test was performed to compare the differential expression levels of PDCD1, CD274, PDCD1LG2, CTLA4, CD276, CD80, CD86, VTCN1, and the TMB between the two risk groups.

### Establishment of a Predictive Nomogram

Using the TCGA training set, a nomogram integrating the IRG signature and stage to predict individual survival was established. In addition, calibration curves and the area under the curve (AUC) for the OS probability at 1, 3, 5 years were plotted to evaluate the predictive accuracy of this nomogram in the TCGA set and the GEO validation set.

### Statistical Analyses

Continuous data are shown as the mean ± SD and were compared using Student’s *t*-test. Categorical variables were analyzed using the chi-square (χ^2^) test. Cox regression analyses were performed to determine the independent prognostic factors for OS. A prognostic nomogram model was established using the “rms” R package, while its predictive accuracy was assessed via the creation of calibration curves. Statistical analysis was performed using SPSS (version 22.0) and R software (version 4.0.1). The threshold of statistical significance was set at a *p*-value < 0.05.

## Results

### Identification of a Prognosis-Related IRG Signature

**FIGURE 1:**
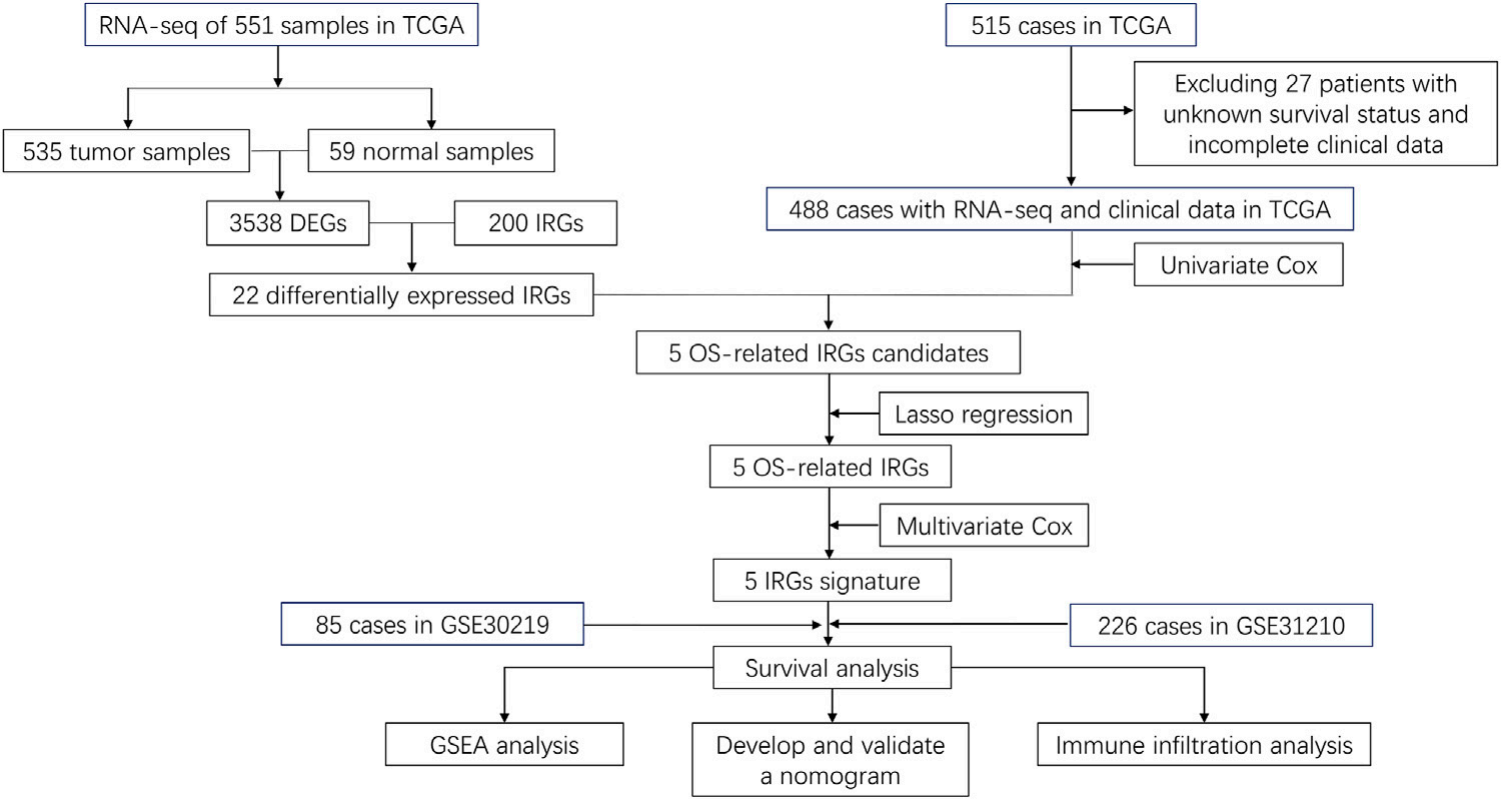
Flow chart of data collection and analysis.

After differential expression analysis in the TCGA dataset between 535 tumor tissues and 59 normal tissues, we discovered 2,849 upregulated and 689 downregulated DEGs, including 22 differentially expressed IRGs. After excluding 27 cases with unsatisfied follow-up or those lacking important clinical information, 488 cases from the TCGA training set were included to identify prognosis-related IRGs and to construct an IRG-based signature. In addition, 226 cases from the GSE31210, and 85 cases in GSE30219 datasets were used to validate the IRG-based signature (Figure 1). The clinicopathological factors of the three datasets are shown in Table 1.

**TABLE 1:** Patients’ characteristics.

	TCGA training cohort *n* = 488	GSE31210 cohort *n* = 226	GSE30219 cohort *n* = 85
Gender			
Male	223 (45.7)	105 (46.5)	66 (77.6)
Female	265 (54.3)	121 (53.5)	19 (22.4)
Age (year)	65.2 ± 10.0	59.6 ± 7.4	61.5 ± 9.3
Smoking history			
No	68 (13.9)	114 (50.4)	0 (0)
Yes or ever	406 (83.2)	112 (49.6)	85 (100)
Unknown	14 (2.9)	0 (0)	0 (0)
Stage			
I	262 (53.7)	168 (74.3)	81 (95.3)
II	121 (24.8)	58 (25.7)	3 (3.5)
III	80 (16.4)	0 (0)	0 (1.2)
IV	25 (5.1)	0 (0)	0 (0)

**FIGURE 2:**
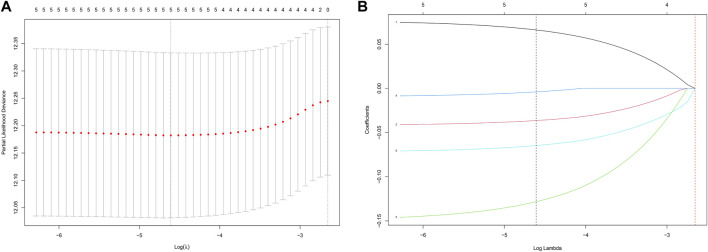
Identification of a prognosis-related IRG-based signature in the TCGA training cohort. **(A)** Selection of the optimal candidate genes in the LASSO model. **(B)** LASSO coefficients of prognosis-associated ARGs, each curve represents a gene.

After univariate Cox analysis using the gene expression profiles of each differentially expressed IRG, five OS-related IRGs from the TCGA training cohort were found (Supplementary Figure S1). These five IRGs were also found to be significant in the LASSO regression analysis (Figures 2A,B), which became the IRG signature candidates. Multivariate Cox regression analysis determined the corresponding regression coefficients of each candidate in this ARG risk signature. Finally, a five-IRG risk signature was constructed according to the 488 LUAD cases in the TCGA cohort, whose risk scores were specifically calculated based on a linear combination of gene expression levels and their corresponding regression coefficients. The specific formula for this is as follows: Risk score = EREG × 0.076861—GPC3 × 0.042023—IL7R × 0.150135—LAMP3 × 0.009471—NMUR1 × 0.072508.

### Prognostic Value of the IRG Signature in the Training Cohort

**FIGURE 3:**
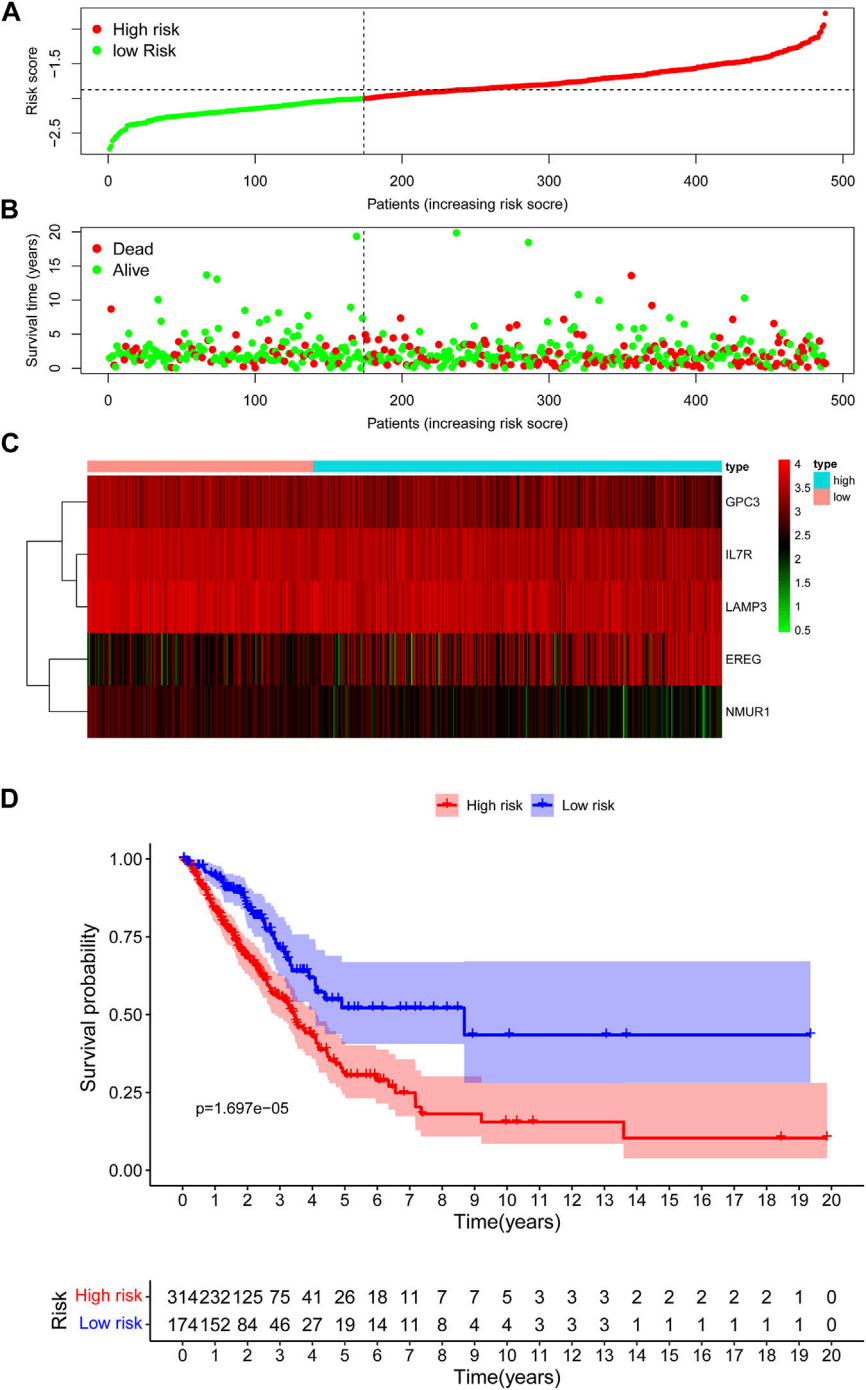
Assessment of prognostic value of the IRG signature model in the TCGA training cohort. **(A)** The distribution of risk scores in the TCGA. **(B)** Patient distribution in the high- and low-risk group according to overall survival status. **(C)** The heatmap showing expression profiles of the five IRGs. **(D)** Kaplan-Meier curves for the overall survival of patients in the high- and low-risk group.

In the TCGA training cohort, the cut-off value of risk scores was determined as -2.0 using the maximally selected log-rank statistics **(** Supplementary Figure S2 **)** after dividing the cases into low-risk and high-risk groups. Figure 3A shows the distribution of the risk scores. As shown in Figure 3B, the high-risk group has significantly more deaths than the high-risk group. A heatmap showing the differential expression of these 5 IRGs between the low-risk and high-risk groups is depicted in Figure 3C. The adjusted FDR and log2 |fold change| of these five IRGs were shown in Supplementary Table S2. Additionally, patients in the low-risk group have significantly longer OS time compared with the high-risk group (*p* = 1.697e−05) **(** Figure 3D **)**.

### Prognostic Value of the IRG Signature in the Validation Cohort

**FIGURE 4:**
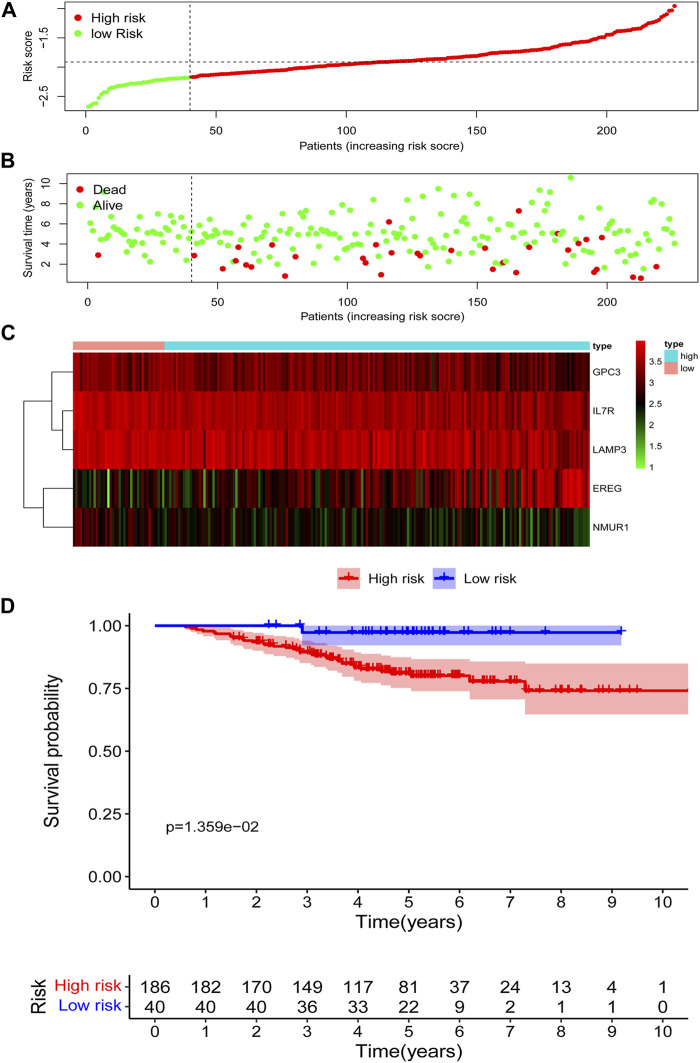
Assessment of prognostic value of the IRG signature model in the GSE31210 validation cohort. **(A)** The distribution of risk scores in the GSE31210 validation cohort. **(B)** Patient distribution in the high- and low-risk group according to overall survival status. **(C)** The heatmap showing expression profiles of the five IRGs. **(D)** Kaplan-Meier curves for the overall survival of patients in the high- and low-risk group.

According to the risk score based on the maximally selected log-rank statistics, 226 cases were divided into the high- (*N* = 186) and low-risk (*N* = 40) groups in the GSE31210 validation cohort. The distribution of risk scores is presented in Figure 4A. Similar to the training cohort, more patients died in the high-risk group compared with the low-risk group **(** Figure 4B **)**. As shown in Figure 4C, the expression profiles of the five IRGs between the low-risk and high-risk groups were plotted in the heatmap and the adjusted FDR and log2 | fold change | of these five IRGs were shown in Supplementary Table S2. The survival curves showed that patients in the low-risk group had a better OS than patients in the high-risk group (*p* = 1.359e−02) (Figure 4D).

**FIGURE 5:**
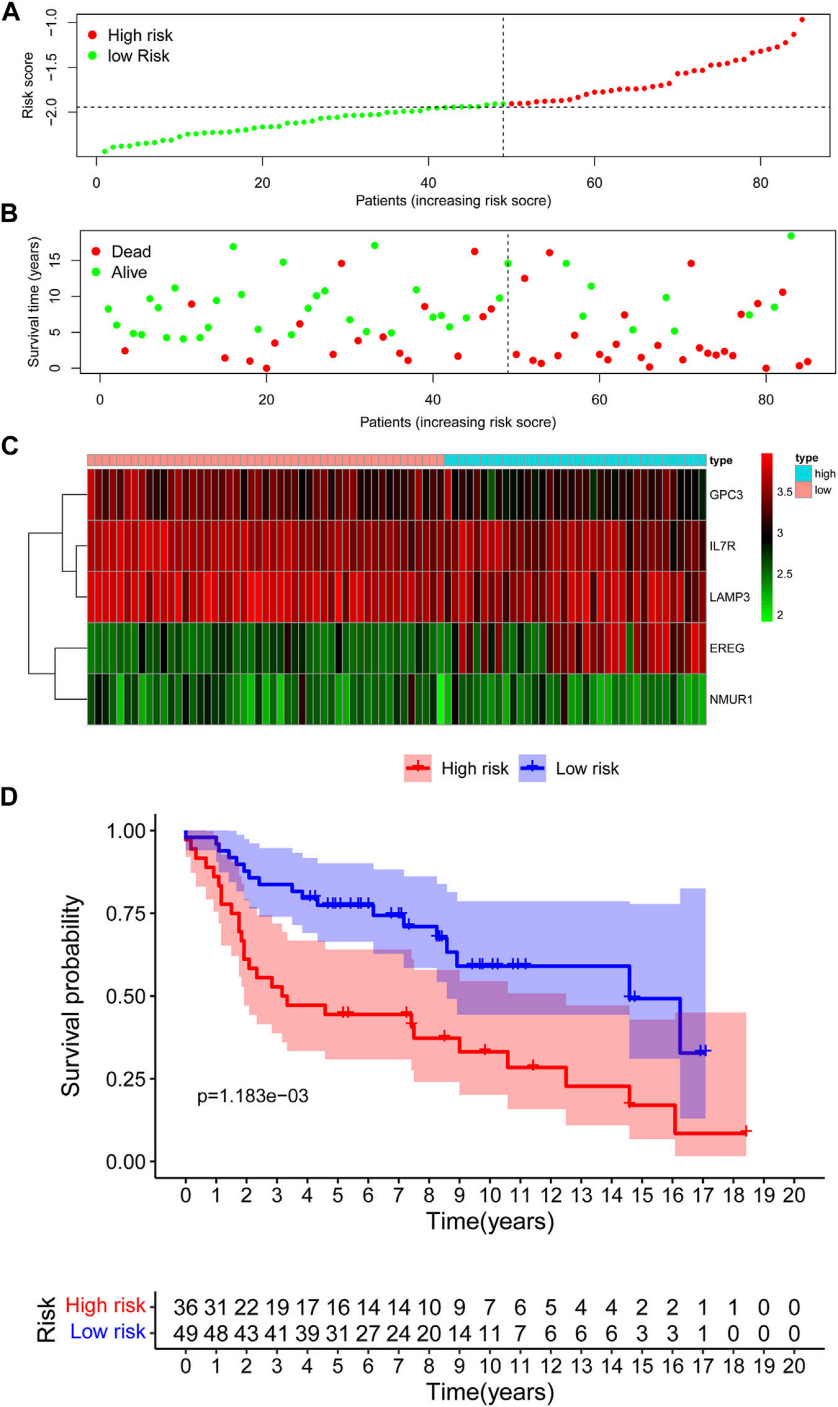
Assessment of prognostic value of the IRG signature model in the GSE30219 validation cohort. **(A)** The distribution of risk scores in the GSE30219 cohort. **(B)** Patient distribution in the high- and low-risk group according to overall survival status. **(C)** The heatmap showing expression profiles of the five IRGs. **(D)** Kaplan-Meier curves for the overall survival of patients in the high- and low-risk group.

The maximally selected log-rank statistics divided 85 cases in GSE30219 validation cohort into high- (*N* = 36) and low-risk (*N* = 49) groups. The distribution of risk scores is presented in Figure 5A. Similar to the training cohort, more patients died in the high-risk group compared with the low-risk group **(** Figure 5B **)**. As shown in Figure 5C, the expression profiles of the five IRGs between the low-risk and high-risk groups were plotted in the heatmap and the adjusted FDR and log2 | fold change | of these five IRGs were shown in Supplementary Table S2. The survival curves showed that patients in the low-risk group had a better OS than patients in the high-risk group (*p* = 1.183e−03) (Figure 5D).

### Gene Set Enrichment Analysis for Important Pathways

**FIGURE 6:**
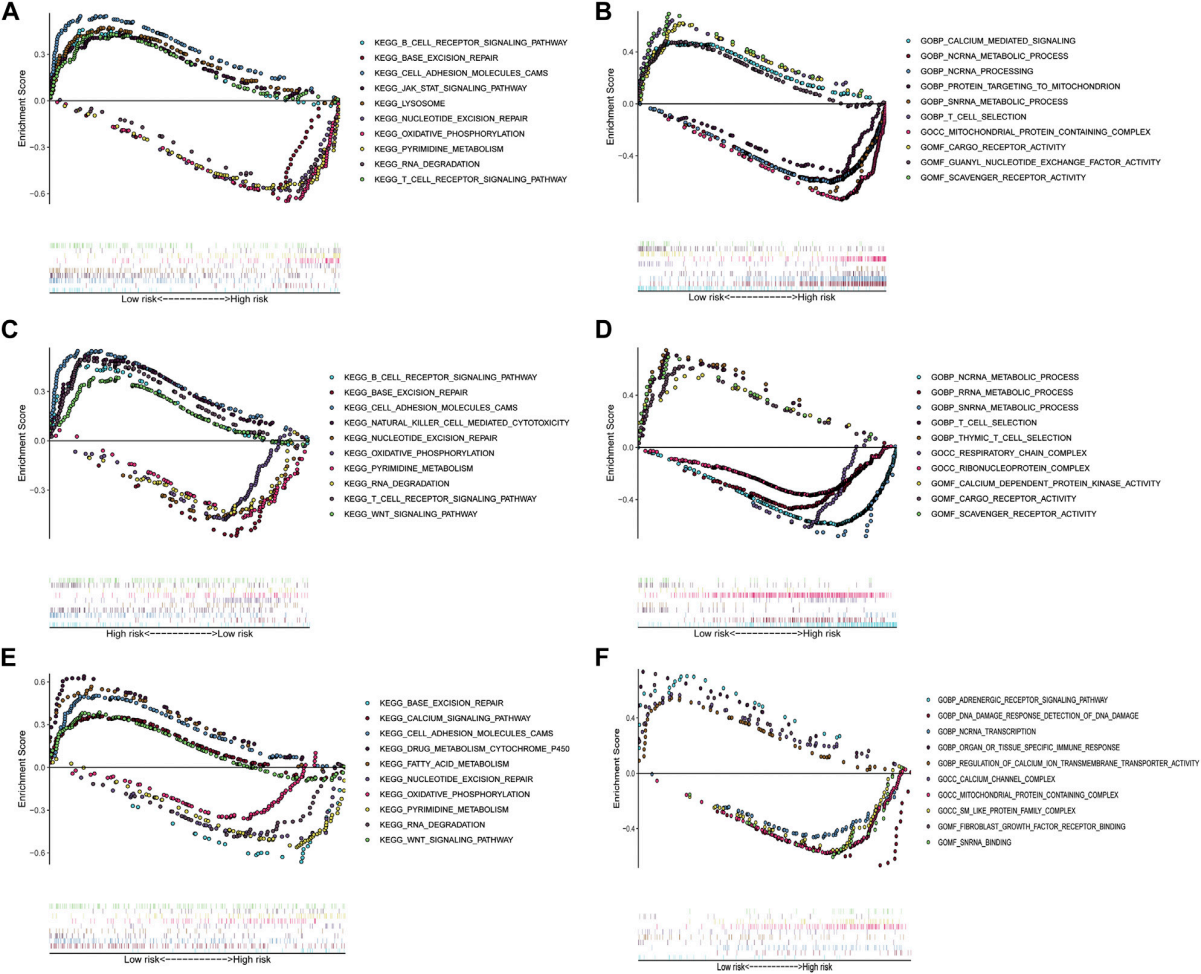
Gene set enrichment analysis between the low- and high-risk subgroups in TCGA training cohort and two GEO validation cohorts. **(A)** Enriched KEGG terms between high- and low-risk groups in TCGA cohort. **(B)** Enriched GO pathways between high- and low-risk groups in TCGA cohort. **(C)** Enriched KEGG terms between high- and low-risk groups in GSE31210 cohort. **(D)** Enriched GO pathways between high- and low-risk groups in GSE31210 cohort. **(E)** Enriched KEGG terms between high- and low-risk groups in GSE30219 cohort. **(F)** Enriched GO pathways between high- and low-risk groups in GSE30219 cohort.

To investigate the underlying functional mechanisms associated with these five IRGs in patients with LUAD, GSEA through GO and KEGG pathway enrichment analysis was performed between the high-risk and low-risk groups in the TCGA training set and two GEO validation set. In the high-risk group, KEGG enrichment analysis found that genes were primarily enriched in base excision repair, nucleotide excision repair, oxidative phosphorylation, pyrimidine metabolism, and RNA degradation in all three datasets. GO enrichment analysis found that genes were primarily enriched in metabolism and function of ncRNA, such as ncRNA metabolic process, ncRNA processing and ncRNA transcription; metabolism and function of scRNA, such as snRNA metabolic process and snRNA binding. In patients with low-risk, KEGG enrichment analysis found that genes were primarily enriched in B cell receptor signaling pathway, cell adhesion molecules cams, T cell receptor signaling pathway, and WNT signaling pathway. GO enrichment analysis found that genes were primarily enriched in metabolism and function of calcium ion, such as calcium-mediated signaling, calcium channel complex, calcium dependent protein kinase activity; T cell selection; cargo receptor activity; and scavenger receptor activity (Figures 6A–F).

### Tumor Immunity Landscape and TMB in LUAD

**FIGURE 7:**
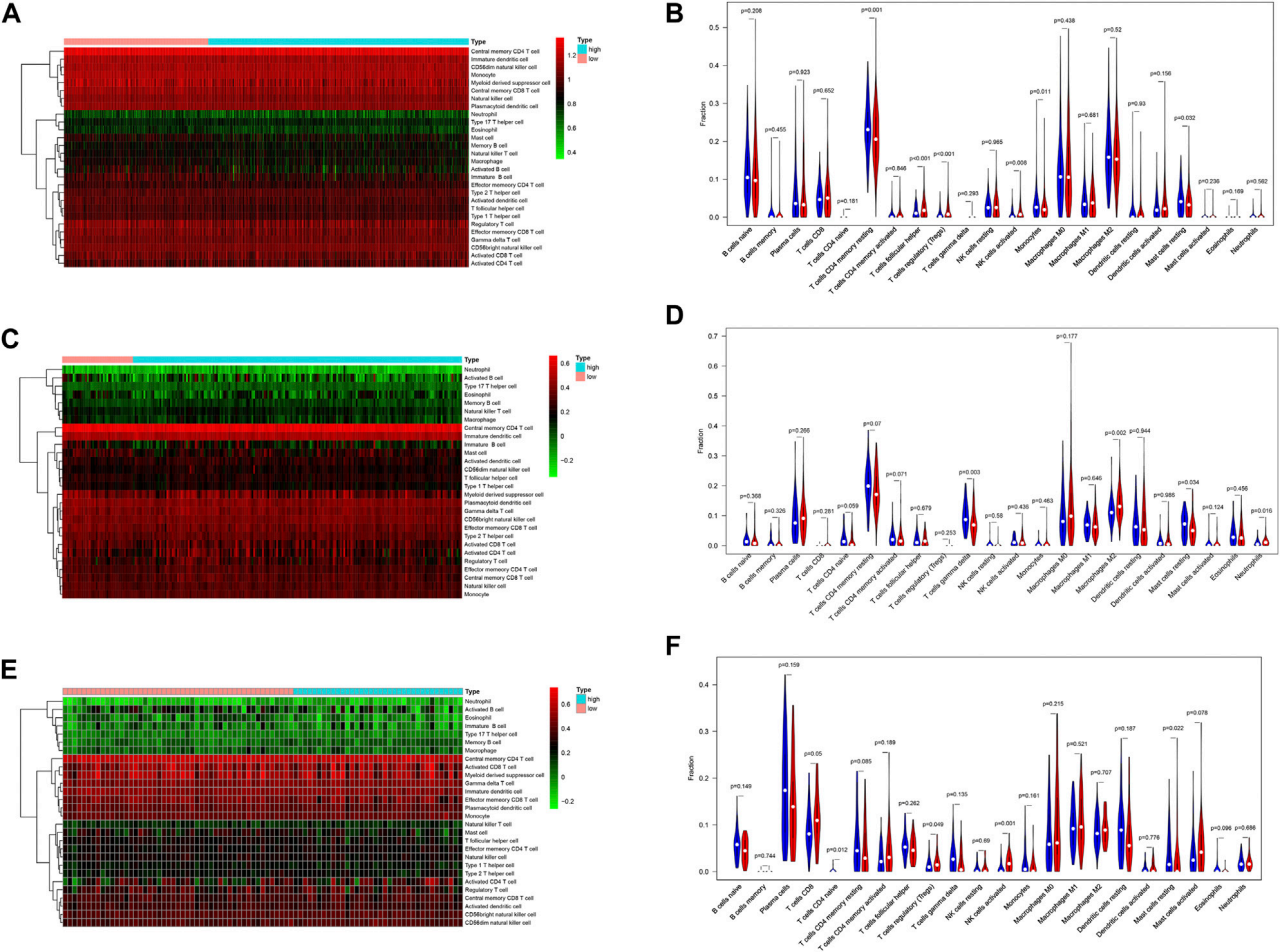
The difference of immune cell infiltration between the high- and low-risk group in the TCGA training and two GEO validation cohort. **(A)** Heatmap of the 28 immune-infiltrating cells landscape of ssGSEA in TCGA cohort. **(B)** Violin plot showing differences of infiltrating immune cell types between the low- and the high-risk group of CIBERSORT in TCGA cohort. **(C)** Heatmap of the 28 immune-infiltrating cells landscape of ssGSEA in GSE31210 cohort. **(D)** Violin plot showing differences of infiltrating immune cell types between the low- and the high-risk group of CIBERSORT in GSE31210 cohort. **(E)** Heatmap of the 28 immune-infiltrating cells landscape of ssGSEA in GSE30219 cohort **(F)** Violin plot showing differences of infiltrating immune cell types between the low- and the high-risk group of CIBERSORT in GSE30219 cohort.

**FIGURE 8:**
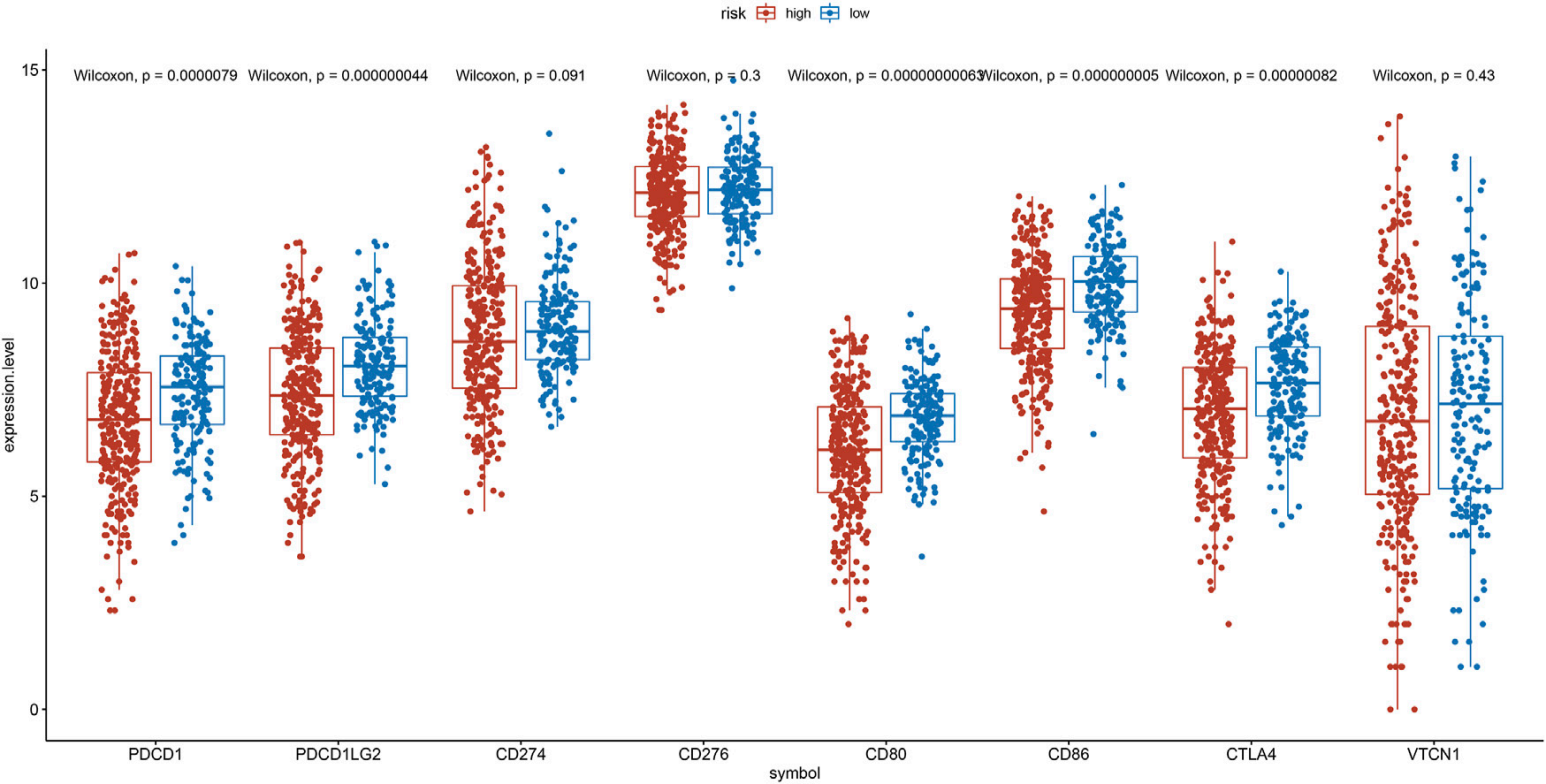
Expression of the PDCD1, PDCD1LG2, CD274, CD276, CD80, CD86, CTLA4, and VTCN1 between the low- and the high-risk group.

To explore the relation between the IRG risk signature and the tumor immunity landscape, the CIBERSORT algorithm and ssGSEA were utilized to evaluate immunity infiltration between low- and high-risk groups in the TCGA training set and 2 GEO validation set. The heatmaps show the immune cell infiltration landscape of the 28 immune cells in three cohorts, as obtained from the results of the ssGSEA analysis (Figures 7A,C,E). The barplot show the immune cell infiltration landscape of the 22 immune cells as obtained from the results of the CIBERSORT algorithm (Supplementary Figure S3A,C,E). As shown in Figures 7B,D,F, patients in low-risk had significantly higher proportions of infiltrating resting mast cells in all three cohorts. Patients in low-risk also had higher proportions of infiltrating resting CD4^+^ memory T cells and monocytes in TCGA cohort, and gamma delta T cell in GSE31210 cohorts. Patients in high-risk group had higher proportions of infiltrating of Treg cells, activated NK cells in TCGA and GSE30219 cohort, while those of follicular helper T cells were significantly higher in TCGA cohort. High-risk patients in GSE31210 had higher proportions infiltrating of M2 macrophage and neutrophils. The proportions of infiltrating 28 immune cells in ssGSEA were shown in Supplementary Figure S3B,D,F. The proportions of infiltrating eosinophils and mast cells were higher in low-risk group among three cohort. In the cohort with large samples, TCGA and GSE31210, low-risk group had higher proportions infiltrating of activated CD8 T cells, effector memory CD8 T cell, central memory CD4 T cell, type 1 helper T cell and activated B cell, which play an important role in anti-tumor immunity. Somatic mutation analysis revealed that patients in the low-risk group had a lower TMB. (Supplementary Figure S4). We also investigated the expression levels of eight immune checkpoint genes between the low- and high-risk groups. There was no difference in the expression level of CD274, CD276, and VTCN1 (Figure 8) between the two risk groups. However, patients in the low-risk group had a higher expression level of PDCD1, PDCD1LG2, CD80, CD86, and CTLA4 (Figure 8).

### Nomogram Based on the IRG Signature for LUAD

**FIGURE 9:**
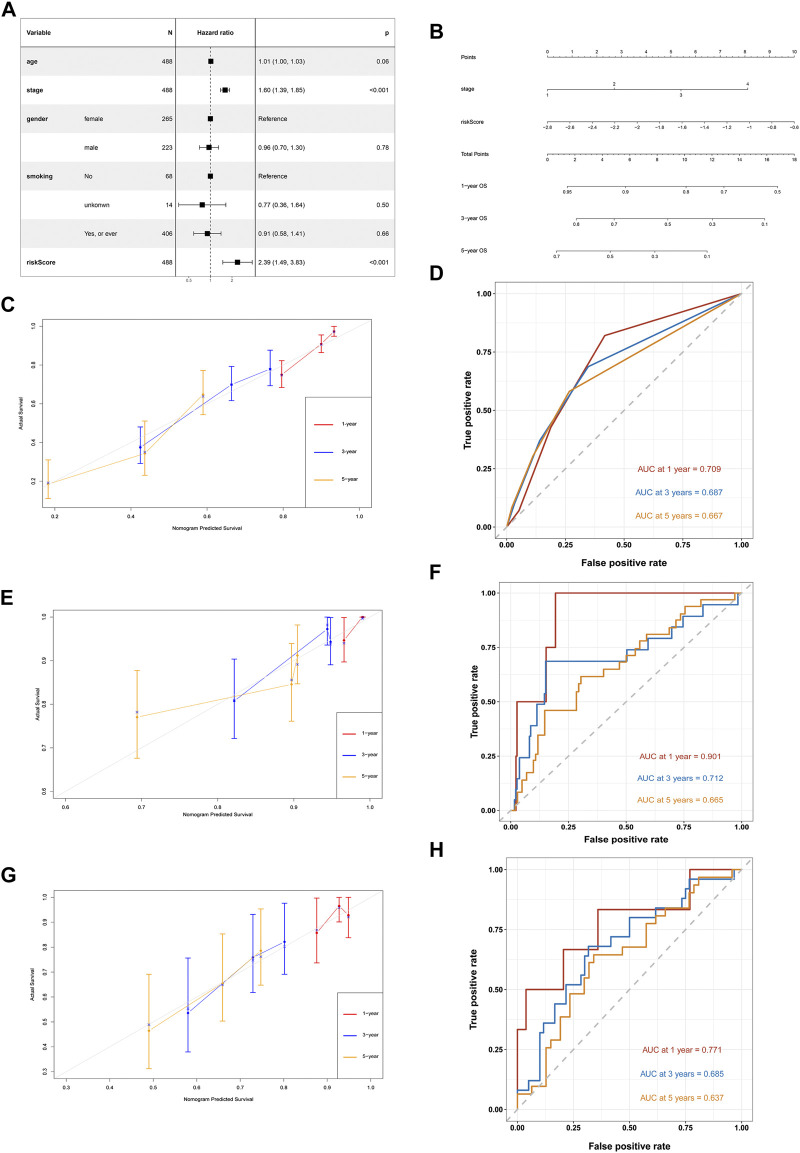
Development of a nomogram based on IRGs signature for predicting overall survival of patients with LUADs. **(A)** Multivariate Cox regression analysis of ARGs signature and other clinicopathological factors. **(B)** The nomogram plot integrating IRG risk score, and stage. **(C)** The calibration plot for the probability of 1-, 3-, and 5-years OS in the TCGA training cohort **(D)** Time ROC curves nomogram-based OS prediction in the TCGA training cohort. **(E)** The calibration plot for the probability of 1-, 3-, and 5-years OS in the GSE31210 cohort. **(F)** Time ROC curves nomogram-based OS prediction in the GSE31210 cohort. **(G)** The calibration plot for the probability of 1-, 3-, and 5-years OS in the GSE30219 cohort. **(H)** Time ROC curves nomogram-based OS prediction in the GSE30219 cohort.

After adjusting for gender, age and smoking history, multivariate Cox analysis demonstrated that the stage (HR = 1.60, 95%CI = 1.39–1.85, and *p <* 0.001) and risk score was a negative prognostic factor of OS in the training cohort (HR = 2.39, 95%CI = 1.49–3.83, and *p <* 0.001) (Figure 9A)**.** Based on the IRG risk scores and stage, a visualized predictive nomogram for individual OS probability at 1, 3, and 5 years was developed using the data of the training cohort (Figure 9B). Bootstrap validation was performed in this nomogram. The C-index of the training cohort was 0.687 (95% CI: 0.644–0.730), while the C-index of the GSE31210 and GSE30219 cohort was 0.678 (95% CI: 0.586–0.771), and 0.656 (95% CI: 0.571–0.740), respectively, which suggested its good performance in predicting OS for LUAD. Calibration curves were drawn in the training and two GEO validation cohorts (Figures 9C,E,G) to verify the accuracy of the nomogram. At 1, 3, and 5 years, the calibration curves demonstrated a satisfactory consistency between the actual observations and the predicted survival probabilities. The time ROC curve in 1, 3, and 5 years also showed the good performance of this nomogram. The AUC values of the 1-, 3-, and 5-years OS in the TCGA cohort were 0.745, 0.690, and 0.697, respectively (Figure 9D). The AUC values of the 1-, 3-, and 5-years OS in the GSE31210 cohort were 0.901, 0.712, and 0.665, respectively (Figure 9F). The AUC values of the 1-, 3-, and 5-years OS in the GSE30219 cohort were 0.771, 0.685, and 0.637, respectively (Figure 9H).

## Discussion

In this study, we explored the relationship between the expression level of IRGs and survival from LUAD. We constructed a novel prognostic ARG signature consisting of five IRGs, including EREG, GPC3, ILR7, LAMP3, and NMUR1. In addition, the multivariate Cox analysis confirmed the prognostic value of the IRG signature. Furthermore, we established a nomogram integrating the IRG signature and stage for predicting individual survival and validated its predictive ability in the GSE31210 and GSE30219 datasets. Finally, we also explored the relationship between expression levels of IRGs and immune cell infiltration in LUAD.

With increasing convenience in data collection from open-access public databases, many studies concentrated on the relationship between RNA-seq data of specific gene sets and individual patient outcomes (Wu et al., 2019; Qu et al., 2020; Zhu et al., 2020; Xu and Chen, 2021). These studies were limited to autophagy, aging, and immune infiltration, but most of them lack clinical applications. In addition, the study focused on the prognostic role of the IRGs in LUAD is lacking. Increasing evidence demonstrated the underlying mechanism of the local inflammatory microenvironment and systemic inflammatory response driving tumorigenesis in many cancers, including lung cancer (Gomes et al., 2014; Lucas et al., 2017; Greten and Grivennikov, 2019; Oya et al., 2020). Several previous studies reported the prognostic value of a single inflammatory-related gene, such as BTG2, TNFRSF10B, and IL1B (Landvik et al., 2012; Schabath et al., 2013; He et al., 2015). However, a comprehensive model with multiple genes and a stronger predictive ability is also necessary. In this study, our nomogram showed good prediction performance in both the training and validation sets.

The IRG risk score formula used in this study indicated that a high gene expression of epiregulin (EREG) was unfavorable for individual survival. EREG is a ligand that belongs to the ERBB family. EREG can bind to the ERBB1 and ERBB4 receptors, activating their intrinsic kinase domain and leading to the phosphorylation of specific tyrosine residues in their receptor’s cytoplasmic tail of their receptors (Shelly et al., 1998). The overexpression of EREG is found in LUAD and associated with unfavorable prognoses (Sunaga and Kaira, 2015). Phosphatidylinositol proteoglycan-3 (GPC3) is an extracellular glycoprotein belonging to the heparan sulfate proteoglycan (HSPG) family (Gonzalez et al., 1998). Interestingly, the *GPC3* gene regulates cell proliferation as a tumor suppressor gene in LUAD (Kim et al., 2003) but shows the opposite effect in LUSC (Lin et al., 2012). Interleukin-7 receptor (IL7R) is the receptor of IL-7, and the IL-7/IL-7R interaction seems to have a two-sided effect in lung cancer. On the one hand, IL-7/IL-7R suppresses autophagy by activating the PI3K/Akt/mTOR signaling pathway and promoting lymphangiogenesis (Ming et al., 2009; Jian et al., 2019). On the other hand, IL-7/IL-7R improves chemotherapeutic sensitivity and anti-tumor immunity (Shi et al., 2019; Bednarz-Misa et al., 2021). Lysosome-associated membrane protein 3 (LAMP3) belongs to the LAMP family of proteins, influencing cellular processes such as phagocytosis, aging, and lipid transport. The role of LAMP3 in cancer remains to be elucidated (Alessandrini et al., 2017). Neuromedin U receptor 1 (NMUR1) is one of the receptors of Neuromedin U. A previous study reported that the methylation of the *NMUR1* gene was related to poor survival in patients with head and neck squamous cell carcinoma (Misawa et al., 2020). The potential mechanisms and function of NMUR1 in LUAD had not been reported.

Tumor inflammation is closely associated with immune cell infiltration in the tumor microenvironment, which contributes to immunotherapy response. However, there is no study exploring the relationship between tumor inflammation and immune cell infiltration in LUAD. The ssGSEA and CIBERSORT algorithms were performed to compare tumor infiltration between the two risk groups in this study. We found that patients in high-risk group had higher proportions of immune cells, which against the anti-tumor immunity, such asregulatory T cells, follicular helper T cells, M2 macrophage, and neutrophils. Previous studies had reported that high proportions of these immune cells promote progression and metastasis in NSCLC (Shi et al., 2014; Marshall et al., 2016; Powell and Huttenlocher, 2016; Pritchard et al., 2020). In patients with low-risk group, a higher proportions infiltrating of resting CD4 memory T cells, activated CD8 T cells, effector memory CD8 T cell, central memory CD4 T cell, type 1 helper T cell, activated B cell and resting mast cells were found, and which contribute to the anti-tumor immunity and are positively associated with prognosis (Chamoto et al., 2006; Lange et al., 2019; Han et al., 2020; Xu et al., 2020). Besides, we investigated the expression level of 8 immune checkpoint genes. The proteins of these 8 genes were costimulatory molecules belong to B7-CD28 family members, which closely associated with anti-tumor immunity (Zhang et al., 2020). Previous study mentioned that patients with high expression level of these 8 genes might had better response of immunotherapy (Li et al., 2019). We found cases in the low-risk group had a higher expression level of PDCD1 (PD-1), PDCD1LG2 (PD-L2), CTLA4, CD80 (B7.1), and CD86 (B7.2), which indicate that patients with a low-risk score may benefit more from immunotherapy.

Some limitations should be considered in this study. First, this IRG prognostic model was established through bioinformatics analyses from data available in the TCGA and GEO databases. Hence, the results of this study need further validation from prospective, multicenter trials or experimental data. In addition, this study preliminarily investigated the potential relationship between the IRG risk signature and immune cells infiltration, so further studies are needed to reveal the underlying mechanisms for this. Finally, although the IRG signature and the stage were integrated into our prognostic nomogram, we cannot identify the contribution of each IRG in this signature.

In conclusion, to determine if the IRG risk signature we deduced was related to OS in patients with LUAD, we constructed and validated a prognostic nomogram for LUAD, including the use of the IRG risk signature and stage, and for predicting individual survival. Patients with a low IRG risk score have a higher expression levels of immune checkpoint gene and this IRG risk signature can be a potential indicator of immunotherapy.

## Data Availability

Publicly available datasets were analyzed in this study. This data can be found here: TCGA databases (https://tcga-data.nci.nih.gov/tcga/) and the GEO databases (https://www.ncbi.nlm. nih.gov/geo/).
