# Supplementary figures and images for: A Novel Inflammatory-Related Gene Signature Based Model for Risk Stratification and Prognosis Prediction in Lung Adenocarcinoma
Source: Front Genet. 2022 Jan 5;12:798131. doi: 10.3389/fgene.2021.798131 (PMC8766344; doi:10.3389/fgene.2021.798131)

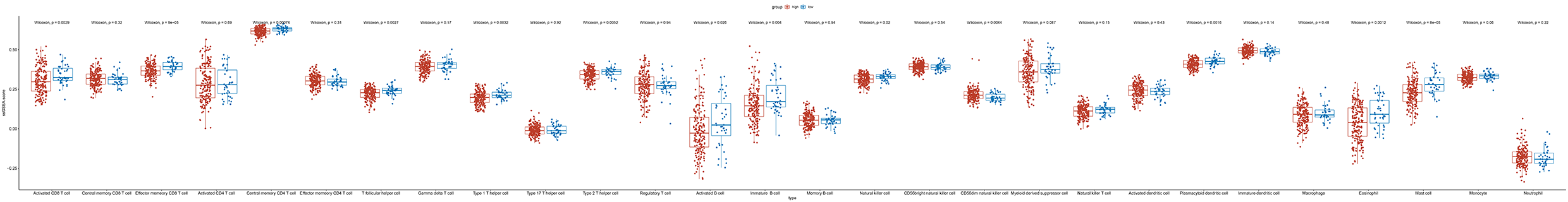

Supplement: Supplementary file 1 [file Image6.TIF]

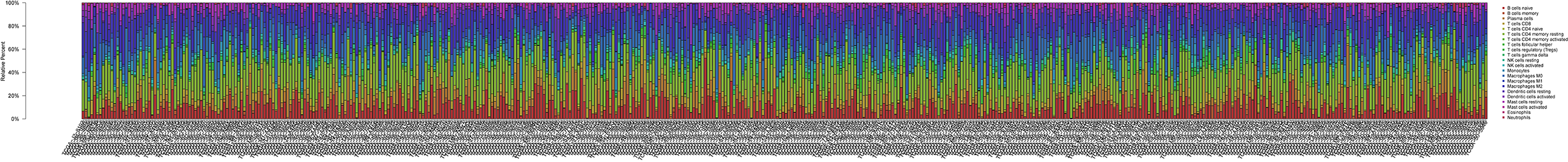

Supplement: Supplementary file 3 [file Image3.TIF]

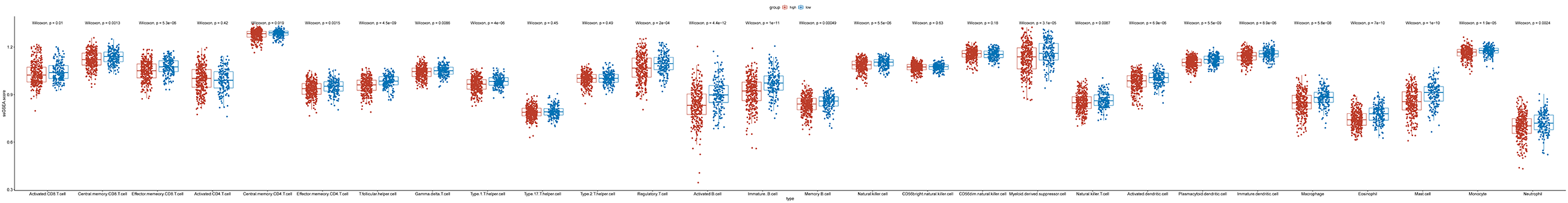

Supplement: Supplementary file 4 [file Image4.TIF]

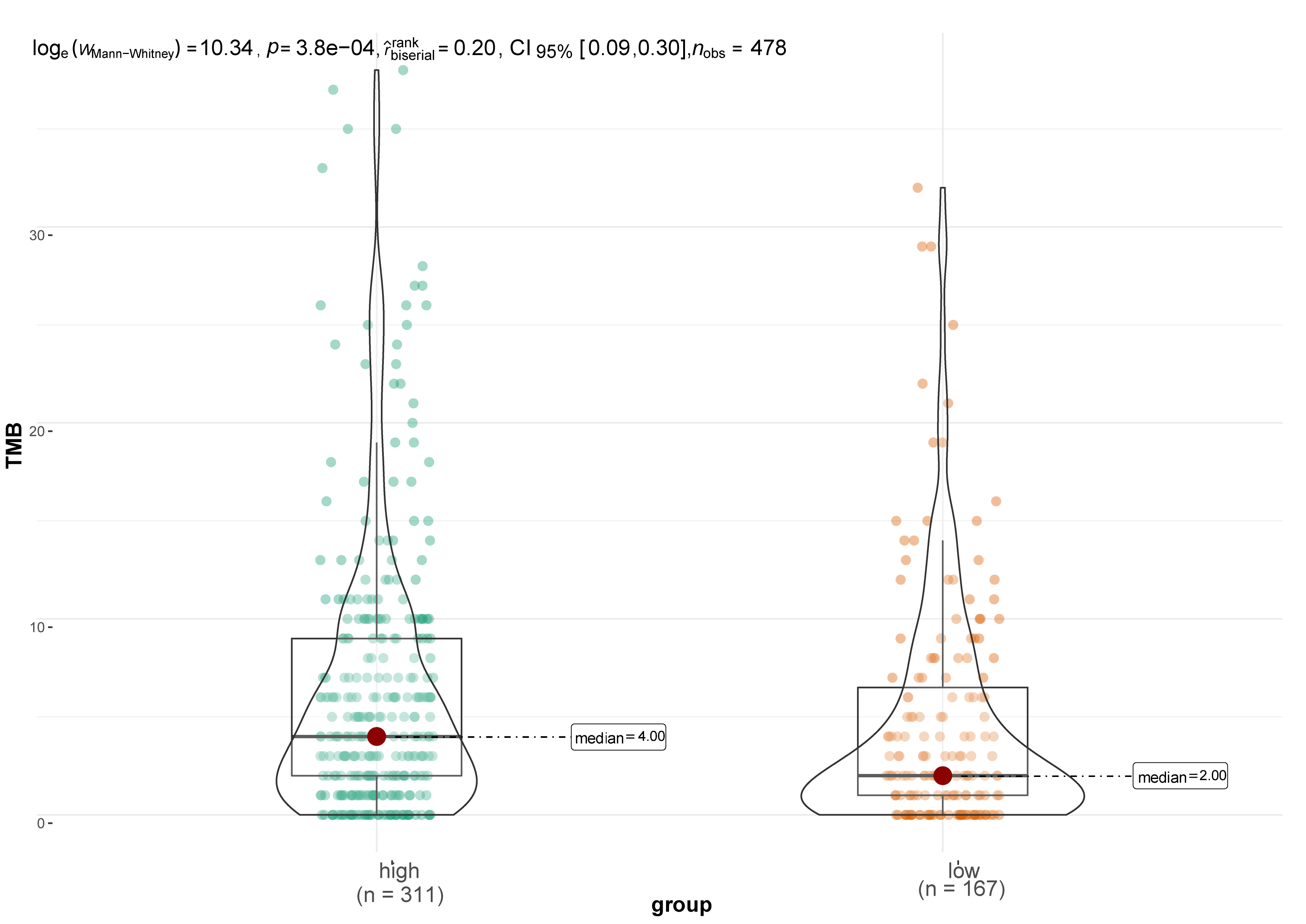

Supplement: Supplementary file 5 [file Image9.TIF]

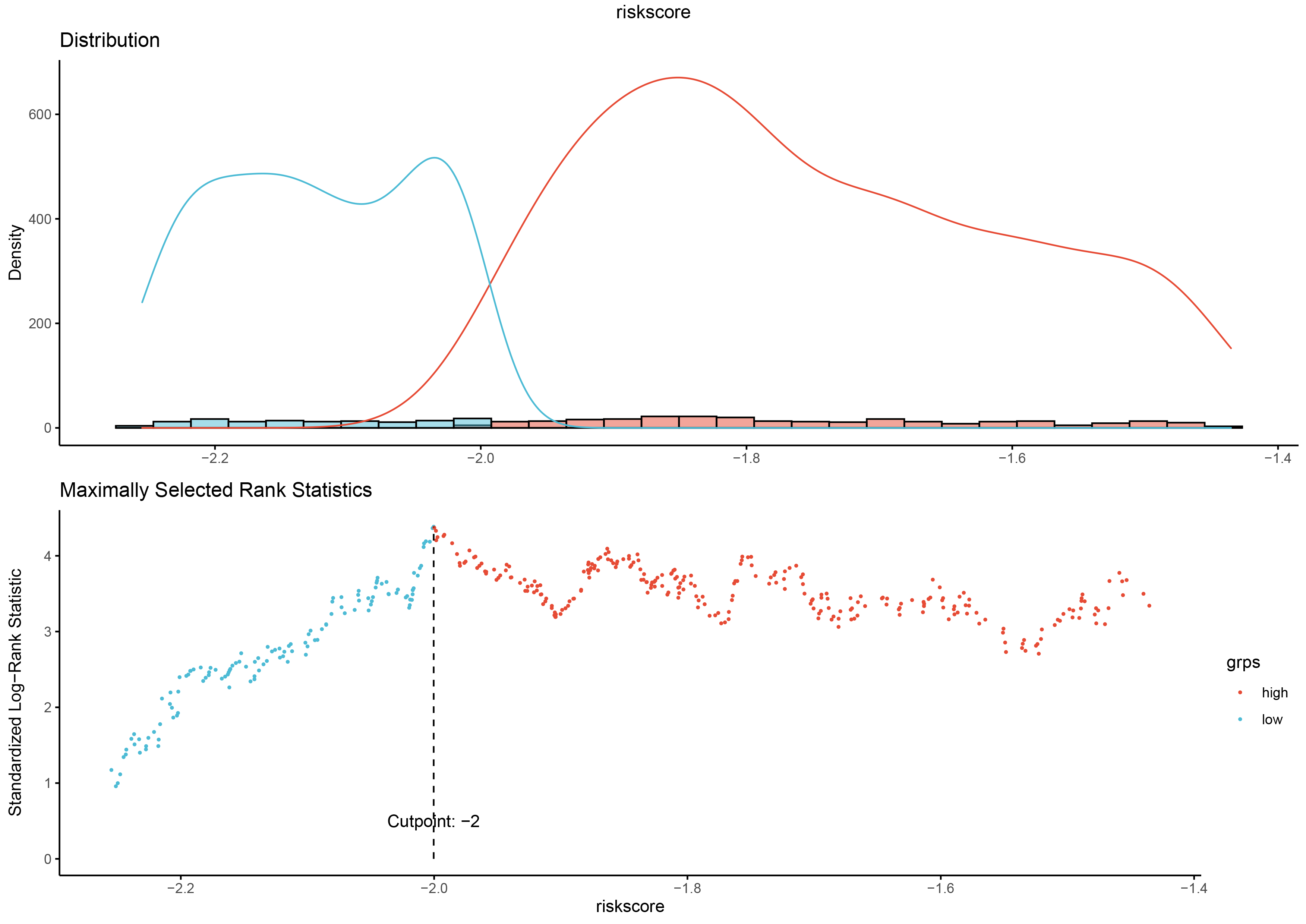

Supplement: Supplementary file 6 [file Image2.TIF]

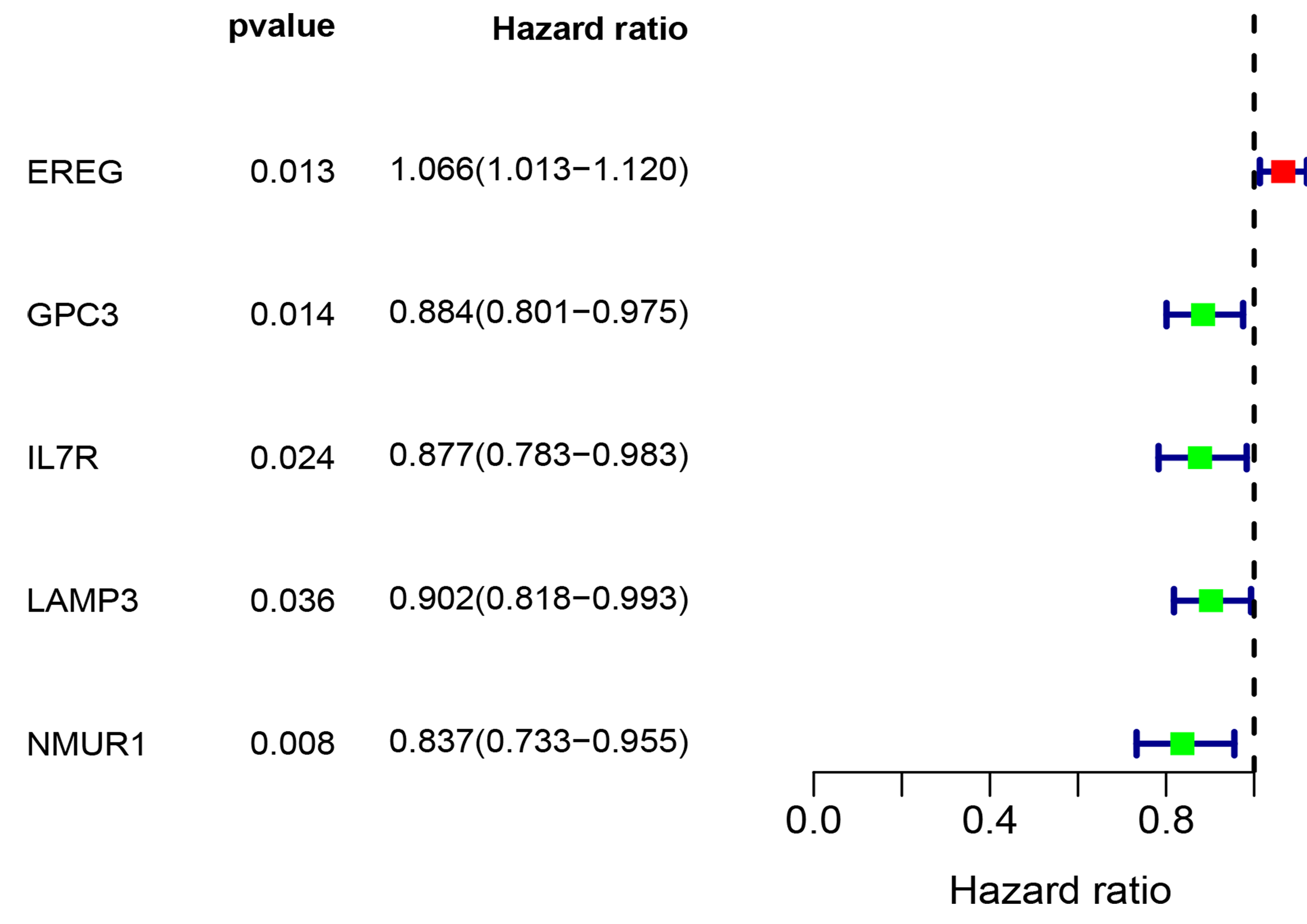

Supplement: Supplementary file 7 [file Image1.TIF]

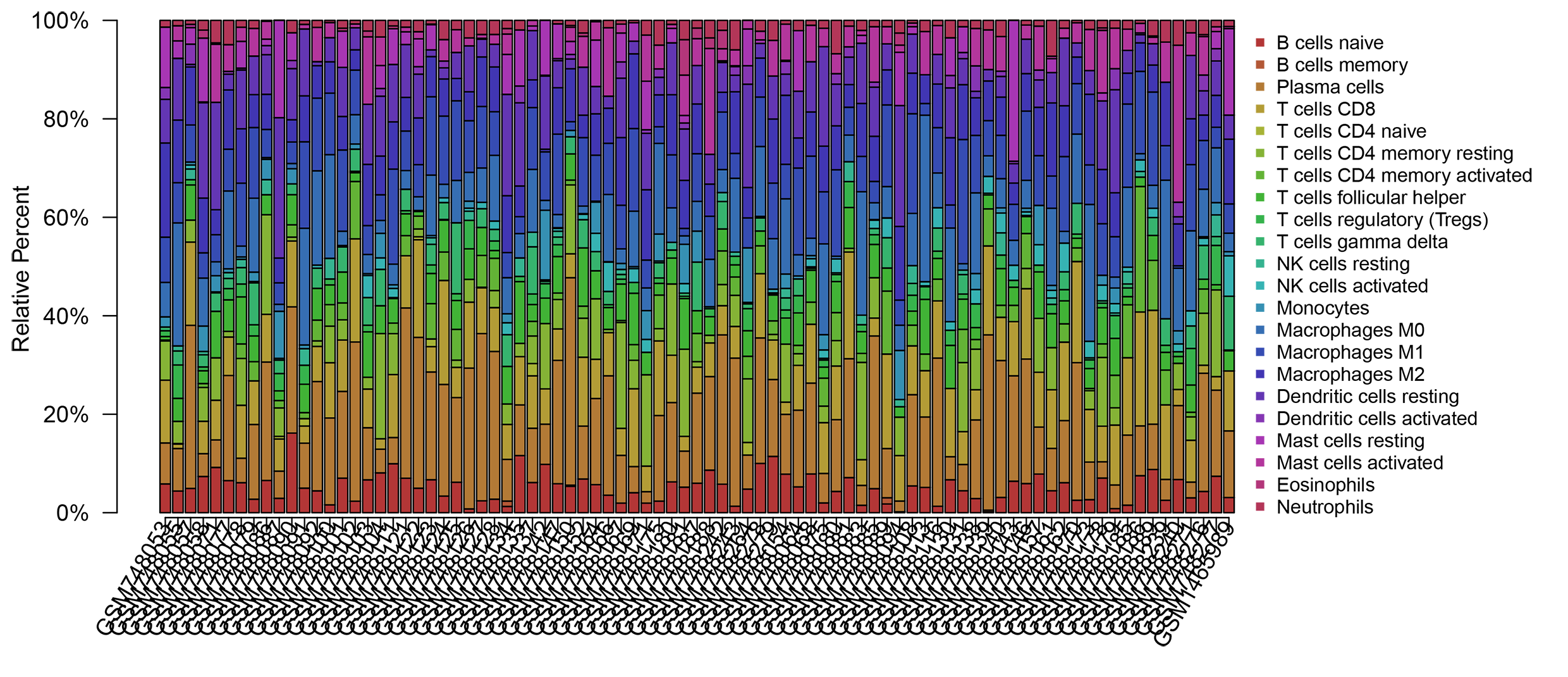

Supplement: Supplementary file 8 [file Image7.TIF]

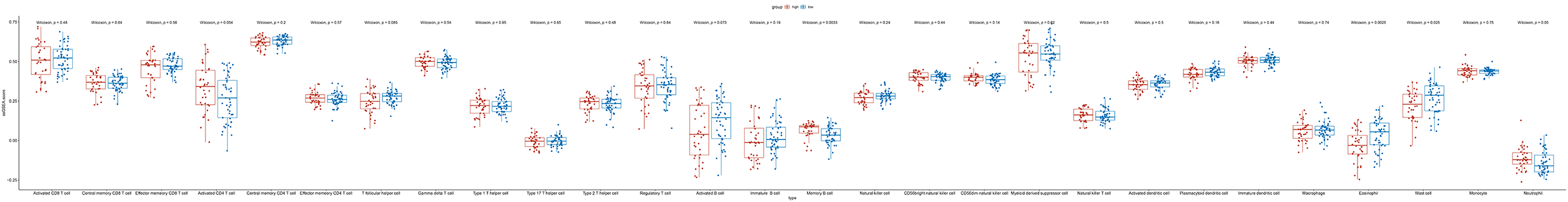

Supplement: Supplementary file 9 [file Image8.TIF]

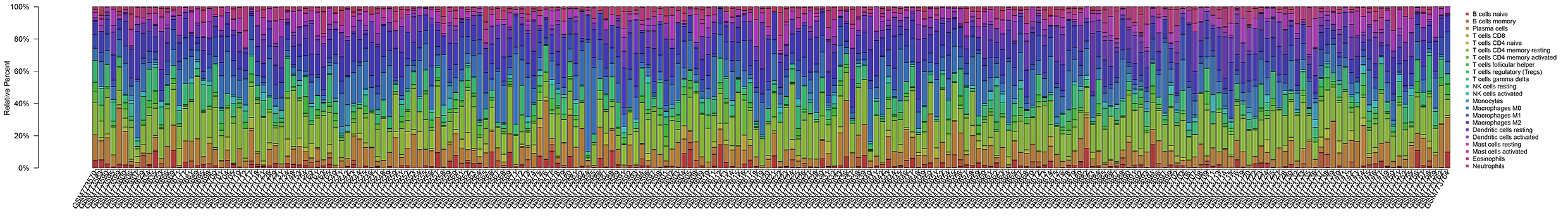

Supplement: Supplementary file 10 [file Image5.TIF]
