# Supplementary material for: A Novel Inflammatory-Related Gene Signature Based Model for Risk Stratification and Prognosis Prediction in Lung Adenocarcinoma
Source: Front Genet. 2022 Jan 5;12:798131. doi: 10.3389/fgene.2021.798131 (PMC8766344; doi:10.3389/fgene.2021.798131)
Supplement: Supplementary file 2 [file Table1.docx]

**Supplementary table Log2 | fold change | and adjusted FDR of 5 IRGs in TCGA, GSE31210 and GSE30219**

|  | Log2FC | Adjusted FDR |
| --- | --- | --- |
| TCGA |  |  |
| EREG | 3.446525 | 8.83E-19 |
| GPC3 | -1.27554 | 3.58E-15 |
| IL7R | -1.01372 | 5.62E-16 |
| LAMP3 | -0.93056 | 3.13E-09 |
| NMUR1 | -1.02916 | 1.36E-17 |
| GSE31210 |  |  |
| EREG | 1.725603 | 0.00167 |
| GPC3 | -0.59438 | 0.006013 |
| IL7R | -1.02116 | 1.48E-09 |
| LAMP3 | -0.87324 | 0.000974 |
| NMUR1 | -0.68687 | 5.25E-05 |
| GSE30219 |  |  |
| EREG | 0.458893 | 1.06E-10 |
| GPC3 | -0.15385 | 0.000626 |
| IL7R | -0.11735 | 0.01307 |
| LAMP3 | -0.1543 | 0.007682 |
| NMUR1 | -0.01497 | 0.585732 |
